# High-depth RNA-Seq data sets to investigate the differences in gene expression mediated by phasevarions in non-typeable *Haemophilus influenzae*

**DOI:** 10.1128/MRA.00785-23

**Published:** 2023-11-22

**Authors:** John M. Atack, Kenneth L. Brockman, Lauren O. Bakaletz, Michael P. Jennings

**Affiliations:** 1 Institute for Glycomics, Griffith University, Gold Coast, Queensland, Australia; 2 School of Environment and Science, Griffith University, Gold Coast, Queensland, Australia; 3 Department of Microbiology and Immunology, Medical College of Wisconsin, Milwaukee, Wisconsin, USA; 4 Center for Microbial Pathogenesis, The Research Institute at Nationwide Children’s Hospital, Columbus, Ohio, USA; 5 The Ohio State University College of Medicine, Columbus, Ohio, USA; University of Southern California, Los Angeles, California, USA

**Keywords:** NTHi, methyltransferase, phase variation, phasevarion

## Abstract

Non-typeable *Haemophilus influenzae* (NTHi) is a major bacterial pathogen of the human airway. We report high-depth coverage RNA-Seq data from prototype NTHi strains 723 and R2866, encoding two of the most common phase-variable ModA alleles found in NTHi strains, ModA2 and ModA10, respectively.

## ANNOUNCEMENT

Non-typeable *Haemophilus influenzae* (NTHi) is responsible for multiple human infections (1–3). NTHi encodes a phase-variable N^6^-adenine DNA methyltransferase (ModA), which regulates genes epigenetically and has key roles in virulence (4–7). Methyltransferase phase variation results in genome-wide methylation differences and regulation of a phasevarion (phase-variable regulon) (8, 9). *modA* alleles have a highly variable (<25% nucleotide-identity) target recognition domain (TRD) that dictates specificity (10). Different TRDs methylate different DNA sequences and control distinct phasevarions (8). Two of the most common *modA* alleles in NTHi are *modA2 and modA10* (5, 11). Using prototype NTHi strain 723 for *modA2* and strain R2866 for *modA10*, we generated locked ON (1 AGCC repeat) or OFF (0 AGCC repeats) variants that could not phase-vary by reducing the number of AGCC_[n]_ repeats in each gene as described previously (12). Triplicate biological replicates of total RNA using Trizol (Thermo Fisher) were prepared according to the manufacturer’s instructions from OD_600_ = 0.5 cultures grown in Brain-Heart Infusion (Oxoid, UK) broth at 37°C with 150 rpm shaking aerobically.

Libraries were prepared using the TruSeq stranded mRNA Kit according to the manufacturer’s instructions (Illumina). As part of this strand-specific protocol, rRNA was depleted using the Ribo-Zero Gold Plus kit (Illumina), and first strand cDNA synthesis (randomly primed) was carried out with SuperScript II Reverse Transcriptase (Invitrogen). Library quality was assessed using an Agilent Bioanalyser DNA 1000 chip. Individual libraries were normalized to 2 nM and pooled using the Illumina cBot system with TruSeq PE Cluster Kit v3 reagents (Illumina). Sequencing was performed on the Illumina NovaSeq system with TruSeq SBS Kit v3 reagents (Illumina; 100 bp paired-end reads for the *modA2* pair; 150 bp paired-end reads for the *modA10* pair). The average number of sequence reads for the triplicate *modA2* locked-ON samples was 46,199,465; for *modA2* locked*-*OFF was 46,780,519, for *modA10* locked*-*ON was 44,449,767, and for *modA10* locked*-*OFF was 44,334,431. Sequence reads were aligned against reference genomes for NTHi strains 723 (accession number CP007472) and R2866 (accession number CP002277) using Bowtie2 aligner (v2.3.3.1) with strand-specific settings. Gene count values were analyzed with edgeR version 3.18.1 (https://bioconductor.org/packages/release/bioc/html/edgeR.html) (13) to compute differential gene expression values with a cutoff with the default TMM normalization method used to normalize the counts between samples. A generalized linear model was then used to quantify the differential expression between the groups, with significant differences between gene expression set as twofold, a count-per-million (CPM) cutoff of 0.5 and a false discovery rate (FDR) cutoff of 0.05. Counts were summarized at the gene level using the featureCounts v1.5.3 utility of the subread package with default settings (https://subread.sourceforge.net/featureCounts.html) (14) for R v3.5.0. Gene expression differences between locked ON-OFF pairs were expressed as logFC (log2-fold change of expression). The analysis generated logCPM values (average log count/million for the gene across all samples), *F* values (quasi-likelihood *F*-statistic for the gene across all samples), *P*-values for the test of statistically different expression, and the FDR (false discovery rate/adjusted *P*-value for multiple hypothesis testing).

By setting a cutoff for greater than twofold differential expression, we show that in strain 723, 88 genes were upregulated, and 88 genes were downregulated, when *modA2* was ON. In strain R2866, 13 genes were upregulated, and 2 genes were downregulated, when *modA10* was ON. These RNA-Seq data sets will serve as an important resource for studying epigenetic gene regulation in NTHi.

## Data Availability

Datasets have been submitted to the NCBI Gene Expression Omnibus (GEO) database and assigned accession numbers GSE106187 (ModA2 ON vs OFF) and GSE129786 (ModA10 ON vs OFF). SRA accession numbers are as follows: for ModA2, SRP121496; for ModA10, SRP192535. These data sets comprise raw reads. Assembled transcripts were not submitted.
